# Perspective on the health effects of unsaturated fatty acids and commonly consumed plant oils high in unsaturated fat

**DOI:** 10.1017/S0007114524002459

**Published:** 2024-10-28

**Authors:** Kristina S. Petersen, Kevin C. Maki, Philip C. Calder, Martha A. Belury, Mark Messina, Carol F. Kirkpatrick, William S. Harris

**Affiliations:** 1 Department of Nutritional Sciences, Pennsylvania State University, University Park, PA, USA; 2 Midwest Biomedical Research, Addison, IL, USA; 3 Indiana University School of Public Health, Department of Applied Health Science, Bloomington, IN, USA; 4 School of Human Development and Health, Faculty of Medicine, University of Southampton, Southampton, UK; 5 Department of Food Science & Technology, The Ohio State University, Columbus, OH, USA; 6 Soy Nutrition Institute Global, Jefferson, MO, USA; 7 Kasiska Division of Health Sciences, Idaho State University, Pocatello, ID, USA; 8 OmegaQuant, Sioux Falls, SD, USA; 9 The Fatty Acid Research Institute, Sioux Falls, SD, USA; 10 Sanford School of Medicine, University of South Dakota, Sioux Falls, SD, USA

**Keywords:** CVD, Seed oils, Linoleic acid, Oxidation, Inflammation, diabetes, n-6

## Abstract

Epidemiological and clinical trial evidence indicates that *n*-6 polyunsaturated fatty acid (PUFA) intake is cardioprotective. Nevertheless, claims that *n*-6 PUFA intake promotes inflammation and oxidative stress prevail. This narrative review aims to provide health professionals with an up-to-date evidence overview to provide the requisite background to address patient/client concerns about oils containing predominantly unsaturated fatty acids (UFA), including MUFA and PUFA. Edible plant oils, commonly termed vegetable oils, are derived from vegetables, nuts, seeds, fruits and cereal grains. Substantial variation exists in the fatty acid composition of these oils; however, all are high in UFA, while being relatively low in saturated fatty acids (SFA), except for tropical oils. Epidemiological evidence indicates that higher PUFA intake is associated with lower risk of incident CVD and type 2 diabetes mellitus (T2DM). Additionally, replacement of SFA with PUFA is associated with reduced risk of CVD and T2DM. Clinical trials show higher intake of UFA from plant sources improves major CVD risk factors, including reducing levels of atherogenic lipids and lipoproteins. Importantly, clinical trials show that increased *n*-6 PUFA (linoleic acid) intake does not increase markers of inflammation or oxidative stress. Evidence-based guidelines from authoritative health and scientific organisations recommend intake of non-tropical vegetable oils, which contain MUFA and *n*-6 PUFA, as part of healthful dietary patterns. Specifically, vegetable oils rich in UFA should be consumed instead of rich sources of SFA, including butter, tallow, lard, palm and coconut oils.

## Graphical abstract

**Figure:**
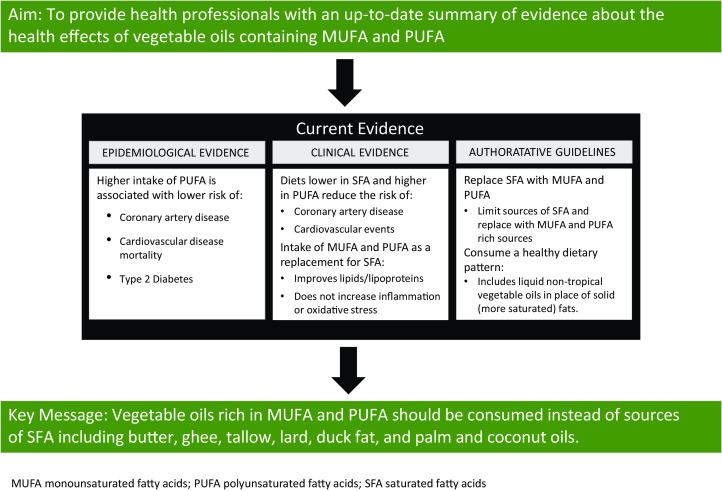

The relationship between dietary fat intake and chronic disease risk, including type 2 diabetes (T2DM)^(1)^, Alzheimer’s disease^(2)^ and certain types of cancer^(3,4)^, has been the subject of considerable investigation with the greatest focus on CVD^(5,6)^. The latter relationship has been investigated since the 1950s^(7)^. However, over the past several decades, a marked shift away from a focus on total fat intake^(8)^ to type of fat^(9)^ has occurred. Contemporary focus is on limiting SFA intake^(10–12)^ and replacing SFA with unsaturated fatty acids (UFA)^(10–12)^, particularly PUFA. This recommendation is based on robust evidence from both observational studies and clinical trials that shows beneficial health outcomes, particularly lower risk of CVD, when SFA are replaced by UFA^(13,14)^. CVD, which includes coronary artery disease (CAD) and cerebrovascular disease (i.e. stroke), continues to be the leading cause of morbidity and mortality in the USA^(15)^ and globally^(16)^.

In the USA, SFA intake generally exceeds recommendations^(17)^. Globally, estimates are that non-optimal intakes of *n*-6 PUFA (<12 % total daily energy [TDE]) and SFA (>10 % TDE) account for 10·3 % and 3·6 % of CAD mortality, respectively^(18)^. However, despite multiple lines of complementary and concordant evidence indicating that *n*-6 PUFA intake is cardioprotective, some authors claim that *n*-6 PUFA intake leads to pro-inflammatory and pro-oxidative states^(19–21)^, which is contributing to a growing social media movement against the use of vegetable oils (also called seed oils)^(22,23)^. The goal of this narrative review is to provide health professionals, especially dietitians and other clinicians, with the information they need to address any concerns their patients/clients may have about oils containing predominantly UFA, including MUFA and PUFA.

As such, the following topics are covered in this review: (1) fatty acid composition of commonly consumed vegetable oils; (2) epidemiological evidence on the association between intake of vegetable oils high in UFA and chronic disease risk with an emphasis on CVD; (3) data from randomised controlled trials (RCT) examining the effect of intake of vegetable oils high in UFA on chronic disease risk with an emphasis on CVD; (4) biological relevance of the dietary *n*-6:*n*-3 fatty acid ratio and (5) guidance for health professionals on vegetable oil use. Non-systematic literature searches were used to identify research on each of these topics. Key conclusions are summarised in Table 1.

**Table 1.** Key conclusions from the evidence reviewed

• Epidemiological evidence shows higher LA intake and circulating levels are associated with lower risk of CVD^(24–26)^ and T2DM^(27,28)^.
• MUFA intake is not consistently associated with CVD risk in epidemiological research^(29)^. Higher intake of MUFA from plant sources is generally associated with lower risk of CVD^(30)^.
• Epidemiological evidence demonstrates higher intake of PUFA, compared to SFA, MUFA or carbohydrates, is associated with reduced risk of CAD^(24,25,31,32)^.
• Evidence from RCT show that diets lower in SFA that include high-PUFA oils reduce the risk of CVD events^(6,33,34)^.
• RCT consistently show improvements in atherogenic lipids and lipoproteins when UFA, particularly PUFA, are consumed instead of SFA^(35)^.
• Evidence from RCT demonstrates that LA intake does not increase inflammation^(36,37)^ or promote oxidative stress^(38,39)^.
• The *n*-6:*n*-3 fatty acid ratio lacks utility because a specific dietary *n*-6:*n*-3 ratio can be achieved by an almost infinite set of dietary patterns, some of which may be deficient in both fatty acid classes. Dietary recommendations should be made based on absolute intake of *n*-3 and *n*-6 PUFA.

## Oil composition

Edible plant oils are commonly referred to as vegetable oils and include oils derived from vegetables, nuts, seeds, fruits and cereal grains. In recent online discussions about oils made from seeds, the term ‘seed oils’ is used^(22,23)^ rather than the more commonly used term ‘vegetable oils’, although ‘seed oils’ has been occasionally used in the scientific literature^(40–43)^. Seed oils include oils derived from sunflower, cottonseed, safflower, canola, sesame, grapeseed, rice bran, soyabean and corn. While the fatty acid composition of these oils varies substantially, all are high in UFA, including MUFA and PUFA, while being relatively low in SFA (Table 2).

**Table 2.** Fatty acid content of selected oils (g/100 g) listed in order of total PUFA content^*^

Oil	Nutrient database number	Total polyunsaturated	Linoleic acid	Alpha-linolenic acid	Monounsaturated	Saturated
Grapeseed	4517	69·9	69·6	0·1	16·1	9·6
Soybean	4044	57·7	51·0	6·8	22·8	15·6
Corn	4518	54·7	53·5	1·2	27·6	12·9
Cottonseed	4502	51·9	55·5	0·2	17·8	25·9
Sesame	4058	41·7	41·3	0·3	39·7	14·2
Rice bran	4037	35·0	33·4	1·6	39·3	19·7
Sunflower^†^	4642	29·0	28·9	<0·1	57·3	9·0
Canola	4582	28·1	19·0	9·1	63·3	7·4
Peanut	4042	19·9	19·6	0·0	57·1	16·2
Avocado	4581	13·5	12·5	1·0	70·6	11·6
Safflower^‡^	4511	12·8	12·7	0·1	75·2	7·5
Olive	4053	10·5	9·8	0·9	73·0	13·8
Palm	4055	9·3	9·1	0·2	37·0	49·3

* USDA FoodData Central, Standard Release Database. https://fdc.nal.usda.gov/.

† Mid-oleic (most commonly used sunflower oil).

‡ High oleic (primary safflower oil of commerce).

**Fig. 1.**
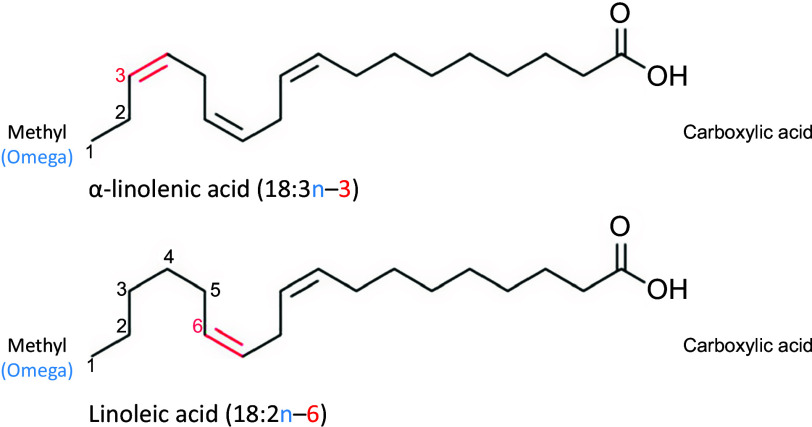
Structure of the two essential fatty acids, linoleic acid and alpha-linolenic acid.

Fatty acids in oils are predominately in the form of triglycerides, which consist of three fatty acids esterified to a glycerol molecule. Fatty acids are comprised of a hydrocarbon chain that varies from four to twenty-four carbons with a methyl group (also known as an omega carbon) at one end and a carboxylic acid group at the other end (Fig. 1). SFA contain no double bonds within the hydrocarbon chain, whereas MUFA contain a single carbon–carbon double bond and PUFA contain at least two carbon–carbon double bonds. The omega system is commonly used to describe the chemical structure of fatty acids with one or more double bonds. In this system, the position of the first carbon–carbon double bond counting from the methyl end of the hydrocarbon chain is used. In the case of *n*-6 and *n*-3 PUFA, the first double bond is at carbon 6 and 3, respectively (Fig. 1).

## Fatty acid intake recommendations

Humans can endogenously synthesise SFA and MUFA in sufficient quantities to meet biological needs. In contrast, humans cannot synthesise the *n*-6 PUFA linoleic acid (LA; 18:2*n*-6) or the *n*-3 PUFA α-linolenic acid (ALA; 18:3*n*-3). Thus, LA and ALA are classified as essential fatty acids and must be consumed in the diet or taken as supplements^(44,45)^. LA deficiency is characterised by rough and scaly skin and dermatitis^(46)^. For LA, the adequate intake established by the USA Institute of Medicine (now the National Academy of Medicine) is 17 g/d and 14 g/d for men 19–50 years and ≥51 years, respectively, and is 12 g/d and 11 g/d for women 19–50 years and ≥51 years, respectively^(47)^. These adequate intakes are based on median intakes in the USA where deficiency is non-existent among healthy individuals. Similarly, adequate intakes for ALA are based on USA median intakes where *n*-3 fatty acid deficiency is non-existent in healthy individuals. The adequate intake for ALA is 1·6 and 1·1 g/d for men and women, respectively^(47)^. The European Food Safety Authority recommendations for LA and ALA are 4 % TDE and 0·5 % TDE, respectively^(48)^. The FAO/WHO recommends 2·5–9 % of TDE be from LA and ALA intake to be >0·5 % TDE for adults^(49)^.

## Oils high in UFA and CVD risk

### Epidemiologic evidence

Evidence from multiple meta-analyses of prospective cohort studies consistently shows that higher PUFA intake (primarily LA) and higher circulating LA levels are associated with lower risk of CVD^(31–33,45)^. Evidence for the relationship between MUFA intake and CVD is less consistent^(29)^, but generally shows higher intake of MUFA from plant sources is associated with lower risk of CVD^(30)^. Findings from meta-analyses demonstrate that higher intake of PUFA, compared with SFA, MUFA, or carbohydrates, is associated with reduced risk of CAD^(24,25,31,32)^.

A meta-analysis of thirteen cohort studies showed that the highest LA intake category was associated with a 15 % lower risk of a CAD event (pooled relative risk [RR] 0·85; 95 % confidence interval [CI] 0·78, 0·92) and a 21 % lower risk of CAD death (RR 0·79; 95 % CI 0·71, 0·89)^(24)^. Similar results were reported by Li *et al.* ^(25)^ in a more recent systematic review and meta-analysis of eighteen prospective cohort studies that examined LA intake. A higher *v*. lower intake of PUFA was associated with a significantly lower risk of CVD mortality (RR 0·87; 95 % CI 0·82, 0·92). It was also found that each 5 % increase in energy intake from LA was associated with a 7 % lower risk of CVD mortality (RR 0·93; 95 % CI 0·91, 0·95)^(25)^.

Circulating LA concentrations reflect dietary intake because LA cannot be endogenously synthesised^(50,51)^. Results from meta-analyses examining the relationship between circulating LA concentrations and CVD are consistent with the results from dietary intake studies^(31,32,45)^. In a meta-analysis of thirty cohort studies (median follow-up 2·5–31·9 years, 15 198 incident cardiovascular events among 68 659 participants), Marklund *et al.* ^(26)^ found that higher circulating LA levels were associated with a significantly lower risk of total CVD (hazard ratio [HR] 0·93; 95 % CI 0·88, 0·99), CVD mortality (HR 0·78; 95 % CI 0·70, 0·85) and ischaemic stroke (HR 0·88; 95 % CI 0·79, 0·98), as well as a nominally lower risk of total CAD (HR 0·94; 95 % CI 0·88, 1·00). Similarly, in a meta-analysis of twenty-two prospective cohort studies that examined LA biomarkers, Li *et al.* ^(25)^ observed that a higher concentration of plasma and tissue LA was associated with an 8 % lower risk of CVD mortality.

Results from pooled analyses and meta-analyses of prospective cohort studies modelling the replacement of SFA with UFA show predicted reductions in risks for CAD events and mortality^(31,32)^. For example, in an analysis of data from three USA cohorts, the Nurses’ Health Study I and II and the Health Professionals Follow-up Study, substitution of 5 % of TDE from SFA with PUFA was associated with a significant reduction in CAD risk (HR 0·75; 95 % CI 0·67, 0·84)^(52)^. Replacement of SFA with MUFA (HR 0·85; 95 % CI 0·74, 0·97) and carbohydrate from whole grains (HR 0·91; 95 % CI 0·85, 0·98) was also associated with significantly lower CAD risk, whereas substitution with carbohydrates from refined starches and added sugars was not^(52)^. Farvid *et al.* ^(24)^ modelled the effect of substituting 5 % of TDE from SFA with LA and found a 9 % lower risk of CAD events (RR 0·91; 95 % CI 0·87, 0·96) and a 13 % lower risk of CAD mortality (RR 0·87; 95 % CI 0·82, 0·94).

It is notable that, after reviewing the available evidence, the 2020 USA Dietary Guidelines Advisory Committee concluded that ‘strong evidence demonstrates that replacing saturated fatty acids with PUFA in adults reduces the risk of CHD events and cardiovascular disease mortality’^(13)^. This conclusion is consistent with evidence-based analyses by other public health and scientific organisations^(10–12,14,33,53–55)^.

### Clinical evidence

Significant clinical evidence supports the beneficial effect of replacing SFA with UFA, particularly PUFA, on cardiovascular events and key risk factors, including lipids/lipoproteins, blood pressure, inflammation and oxidative stress.

In RCT examining diets lower in SFA that included high-PUFA oils, clinically relevant reductions in CVD events have been observed^(6,33,34)^. In the most recent (2020) Cochrane Review, which included thirteen RCT (sixteen comparisons, 53 758 participants, mean duration 4·7 years), reducing SFA intake lowered the risk of combined cardiovascular events by 17 % (RR 0·83; 95 % CI 0·70, 0·98), and the quality of evidence was assessed as moderate^(6)^. Further analyses examining the replacement macronutrient showed that replacing SFA with PUFA was associated with a nominal 21 % (RR 0·79; 95 % CI 0·62, 1·00; eight trials) reduction in the risk of combined cardiovascular events, whereas replacement of SFA with carbohydrates (RR 0·84; 95 % CI 0·67, 1·06; five trials), MUFA (RR 1·00; 95 % CI 0·53, 1·89; one trial) or proteins (RR 0·97; 95 % CI 0·91, 1·03; four trials) was not significantly associated with cardiovascular event risk.

An earlier meta-analysis of RCT (median duration 4·25 years) that included 13 614 participants showed that replacing 5 % of TDE from SFA with equivalent energy from PUFA was associated with a 10 % reduction in CAD risk (RR 0·90; 95 % CI 0·83, 0·97)^(34)^. This effect estimate was similar to the predicted CAD risk reduction based on the total cholesterol to HDL-cholesterol ratio (RR 0·91; 95 % CI 0·87, 0·95), which suggests that improvements in lipids/lipoproteins account for most of the protection against CAD. These findings are consistent with those from a meta-analysis conducted for an American Heart Association Presidential Advisory that included four trials, which provide the core RCT evidence for replacement of SFA with PUFA^(33)^.

These four trials^(56–61)^ provide the core evidence because of the quality of study design, execution and intervention adherence (Table 3). Six trials^(62–67)^ were identified that did not meet the inclusion criteria in Table 3 and, therefore, provide low-quality evidence. A meta-analysis of the four high-quality studies showed that replacement of SFA with PUFA was associated with a 29 % reduction in risk of CAD (RR 0·71; 95 % CI 0·62, 0·81). While there are RCT showing that intake of high-PUFA-containing oils is not associated with reduced risk of CVD^(66,68)^, these have fundamental flaws, which preclude meaningful conclusions^(69)^. Limitations include (1) mixed dietary interventions in which PUFA and carbohydrate replaced SFA; (2) insufficient duration; (3) low adherence and (4) few events. Collectively, the available high-quality RCT evidence shows that replacement of SFA with PUFA-containing oils lowers CVD risk to a magnitude similar to statin therapy^(70)^.

**Table 3.** Criteria for inclusion of RCT in the 2017 American Heart Association Presidential Advisory on dietary fats and CVD

• Compared high SFA with high PUFA intake • Did not include *trans* UFA as a major component of the intervention or control diets • Controlled the dietary intake of the intervention and control groups • Had ≥2 years of sustained intake of the assigned diets • Proved adherence by objective biomarkers, such as serum cholesterol and/or blood or tissue levels of PUFA • Collected and validated information on cardiovascular or coronary disease events

### Oils high in UFA and CVD risk factors

Although it is well established that LDL-cholesterol has a causal role in the development of CVD^(71,72)^, a point of debate among scientists and clinicians is whether diet-related reductions in LDL-cholesterol translate to CVD risk reduction^(73–75)^. A systematic review and meta-analysis of forty-nine RCT of pharmacological and dietary interventions (312 175 participants, 24 % women; mean baseline LDL-cholesterol level of 3·16 mmol/l (122·2 mg/dl), 39 645 major vascular events) showed the RR reduction for major vascular events was proportional to LDL-cholesterol-lowering achieved. Specifically, per 1 mmol/l (39 mg/dl) decrease in LDL-cholesterol, the RR of a major vascular event was reduced by 23 % (RR 0·77; 95 % CI 0·75, 0·79)^(76)^.

It has also been suggested that LDL-cholesterol lowering in response to dietary reductions in SFA does not confer protection against CVD^(72)^ because some evidence shows SFA replacement lowers larger LDL particle subspecies^(77)^, which are not strongly associated with CVD risk^(72)^. Generally, smaller, denser LDL particles are considered more atherogenic^(72)^. However, it is important to note that statins preferentially lower larger LDL particles and significantly lower CVD risk^(78)^. In addition, epidemiological analyses show that the association between LDL particle size or subclass distribution and CVD risk is attenuated to nonsignificance after adjustment for LDL particle concentration^(79)^. Clinically, LDL-cholesterol concentration is used to estimate CVD risk^(80)^ because, under most conditions, LDL-cholesterol concentration is highly correlated with LDL particle number^(71)^.

In most individuals, LDL particles constitute ∼90 % of circulating apolipoprotein B (apoB)-containing lipoproteins in the fasting state^(71)^. Each LDL particle contains one apoB molecule; thus, measurement of the apoB concentration reflects the total circulating concentration of lipoproteins with atherogenic potential. Critically, it is the trapping of apoB-containing lipoproteins in the artery wall that initiates and drives the atherosclerotic process^(81)^. Therefore, examining apoB concentration is informative to CVD risk reduction estimates.

The results of RCT consistently show improvements in atherogenic lipids and lipoproteins when UFA, particularly PUFA, are consumed instead of SFA. A systematic review and meta-regression analysis that included eighty-four RCT (duration ranging from 13 to 91 d) involving 2353 participants showed that when 1 % of TDE from SFA is isocalorically replaced with PUFA (predominately LA), lowering of total cholesterol (mean change –2·47 mg/dl; 95 % CI –2·71, –2·24), LDL-cholesterol (–2·13 mg/dl; 95 % CI –2·36, –1·93), total cholesterol to HDL-cholesterol ratio (–0·034; 95 % CI –0·04, –0·028) and apoB (–10·2 mg/dl; 95 % CI –12·4, –8·1) is observed^(35)^. Similarly, when 1 % of TDE from SFA is isocalorically replaced with MUFA, total cholesterol (–1·78 mg/dl; 95 % CI –1·97, –1·55), LDL-cholesterol (–1·62 mg/dl; 95 % CI –1·82, –1·43), total cholesterol to HDL-cholesterol ratio (–0·027; 95 % CI –0·033, –0·022) and apoB (–7·8 mg/dl; 95 % CI –9·5, –6·0) are reduced. Thus, high-quality evidence supports intake of PUFA and MUFA in place of SFA to improve lipids/lipoproteins.

The effects of fatty acids on CVD risk and lipoprotein levels align with the results of analyses focusing on the intake of specific vegetable oils. For example, systematic reviews and meta-analyses of RCT show intake of canola oil^(82,83)^ and rice bran oil^(84)^ lowers total cholesterol and LDL-cholesterol levels. Similarly, RCT examining soyabean oil^(85–91)^, corn oil^(92–94)^, cottonseed oil^(95,96)^ and sunflower oil^(97,98)^ consistently show lipid/lipoprotein improvements. Finally, in a network meta-analysis of RCT (duration ranging from 3 to 17 weeks) where direct and indirect evidence on the effects of thirteen oils and solid fats (safflower, sunflower, canola, hempseed, flaxseed, corn, olive, soyabean, palm and coconut oils as well as beef fat, lard and butter) was synthesised, it was shown that replacement of 10 % of TDE from butter with an equivalent amount of safflower, sunflower, canola, olive, flaxseed, corn or soyabean oil lowered LDL-cholesterol by 10–16 mg/dl^(99)^. In summary, replacement of dietary sources of SFA with oils rich in PUFA and MUFA consistently improves lipids and lipoprotein levels.

Researchers have also investigated the effects of dietary fatty acids on blood pressure. However, in contrast to the effects on lipids and lipoproteins, RCT generally show replacement of SFA with MUFA or PUFA has little to no impact on this metric^(100–103)^. A 2018 Cochrane Review of RCT comparing higher *v*. lower intake of *n*-6 PUFA for a minimum duration of 12 months found no clinically relevant effects on systolic or diastolic blood pressure, although this finding was based on only two studies^(104)^. In agreement, in the Dietary Intervention and VAScular function study, replacing ∼9·5 % of TDE from SFA with MUFA or *n*-6 PUFA for 16 weeks did not affect 24-hour systolic or diastolic blood pressure in individuals at moderate CVD risk^(103)^. However, replacing SFA with MUFA did reduce nighttime systolic blood pressure (mean difference –4·9 mmHg; *P* = 0·019). Finally, in the OmniHeart RCT, a higher fat, MUFA-rich diet (48 % kcal carbohydrate, 37 % kcal total fat, 21 % kcal MUFA, 10 % kcal PUFA, 6 % kcal SFA) modestly lowered systolic (mean difference –1·3 mmHg, *P* = 0·005) and diastolic (–0·8 mmHg, *P* = 0·02) blood pressure after 6 weeks compared to a carbohydrate-rich diet (58 % kcal carbohydrate, 27 % kcal total fat, 13 % kcal MUFA, 8 % kcal PUFA, 6 % kcal SFA)^(105)^. In summary, evidence from RCT suggests replacing SFA with MUFA may modestly improve blood pressure, whereas PUFA have no effect.

As noted previously, a commonly expressed concern about LA is that it increases chronic inflammation. However, the available evidence does not support this concern. A recent systematic review and meta-analysis that included thirty RCT with a duration of 4 to 24 weeks demonstrated that higher intake of LA did not increase tumor necrosis factor-α (standardised mean difference [SMD] –0·01; 95 % CI –0·19, 0·17), interleukin-6 (SMD 0·11; 95 % CI –0·07, 0·29), adiponectin (SMD 0·17; 95 % CI –0·17, 0·50), monocyte chemoattractant protein 1 (SMD 0·14; 95 % CI –0·33, 0·60), or C-reactive protein (SMD 0·09; 95 % CI –0·05, 0·24)^(36)^. In agreement, after systematically reviewing fifteen RCT (duration 2 to 9 weeks) involving healthy participants, Johnson and Fritsche^(37)^ concluded that there is virtually no evidence to show that addition of LA to the diet increases the concentration of inflammatory markers.

Another concern is that *n*-6 PUFA, including LA, promote oxidative stress^(20,106,107)^. Oxidative stress is defined by a disturbance in the balance between production of reactive oxygen species (free radicals or chemical species that contain unpaired electrons) and antioxidant defenses, which may lead to tissue damage^(108)^. Oxidative stress has been implicated in the development of many chronic diseases, including CVD, cancer, T2DM and neurological diseases^(109)^. PUFA are susceptible to oxidation because they contain multiple double bonds^(110)^. This susceptibility has given rise to the concern that greater intake of PUFA results in PUFA-enrichment of LDL particles, thereby increasing their susceptibility to oxidative modification. This susceptibility has been demonstrated in *in vitro* and *ex vivo* experiments^(111,112)^. However, the results of most RCT show no effect of *n*-6 PUFA on markers of oxidative stress, including oxidised LDL^(38)^ and F-2 isoprostanes, a marker of lipid peroxidation^(39)^. As reviewed by Birben *et al.* ^(113)^, aerobic organisms have integrated antioxidant systems, which include enzymatic and non-enzymatic antioxidants that are effective in blocking harmful effects of reactive oxygen species.

The lack of effect of *n*-6 PUFA on markers of oxidative status is illustrated by research involving soybean oil. Of the four RCT, with a duration of 4–12 weeks, examining the effects of soybean oil (∼51 % LA) with oils lower in LA, only one showed an increase in a marker of oxidative status (decrease in small, dense LDL oxidation lag time)^(89)^. However, given the *in vitro* test used to measure lag time in this study and a substantial reduction in LDL-cholesterol in response to soybean oil intake, the overall health effect is likely beneficial. Furthermore, in two trials, no effects on markers (thiobarbituric acid reactive substances, malondialdehyde, oxidised LDL-cholesterol) of oxidative status were observed^(114,115)^, and, in one trial, trolox equivalent antioxidant capacity actually increased in response to soybean oil^(116)^.

Finally, it is established that chronic hyperlipidaemia promotes oxidative stress, with more pronounced effects in individuals with obesity^(117)^. Therefore, total cholesterol and LDL-cholesterol lowering induced by replacing SFA with UFA, including PUFA, would be expected to reduce oxidative stress.

## UFA and T2DM

### Epidemiologic evidence

The association between PUFA or LA intake and risk of developing T2DM has been examined in numerous cohort studies, especially in Western countries. For example, in the Nurses’ Health Study I, a 5 % increase in energy from PUFA (mainly LA) was associated with a marked lowering of T2DM risk (RR 0·63; 95 % CI 0·53, 0·76)^(118)^. In agreement are the results of another USA study involving 35 988 older women. During the 11-year follow-up period, 1890 women developed T2DM. After adjusting for potential confounders, RRs across increasing quintiles of vegetable fat intake were 1·00, 0·90, 0·87, 0·84 and 0·82 (*P* = 0·02). Lower risk of incident T2DM was also associated with modelled substitution of PUFA with SFA^(119)^.

Consistent with the results of the two studies discussed above are those from the Health Professionals Follow-Up Study, which involved 42 504 men aged 40–75 years at study entry. LA intake was associated with a lower risk of developing T2DM in men < 65 years of age (RR 0·74; 95 % CI 0·60, 0·92) and in those with a body mass index (BMI) < 25 kg/m^2^ (RR 0·53; 95 % CI 0·33, 0·85)^(120)^. However, no relationship was found between LA intake in older men or men with obesity (BMI > 30 kg/m^2^). More recently, a pooled analysis of the Nurses’ Health Study I and II and the Health Professionals Follow-Up Study, which involved over 200 000 USA men and women, showed that dietary LA intake was significantly inversely related to risk of incident T2DM over the nearly 3-decade follow-up period (HR 0·92; 95 % CI 0·87, 0·98)^(121)^.

PUFA intake has also been found to be protective against T2DM in cohorts outside of the USA. For example, in the European Prospective Investigation of Cancer-Norfolk study, the energy-adjusted dietary PUFA:SFA ratio was inversely associated with the risk of T2DM (per standard deviation change, odds ratio 0·84; 95 % CI 0·75, 0·94)^(122)^. However, a later publication from this cohort reported no significant association between dietary LA and incident T2DM^(123)^.

More important than the results of individual studies are the findings of systematic reviews and meta-analyses of epidemiological evidence. These reviews are generally supportive of the benefits of PUFA. For example, the authors of a 2014 meta-analysis of four cohort studies concluded that an increase in PUFA intake, mainly *n*-6 PUFA, from 3 % to ∼6 % of energy in exchange for carbohydrate or SFA may be associated with a 20 % reduction in T2DM risk^(27)^. They also noted that tissue LA is inversely associated with the development of T2DM. These results align with a more recent systematic review and meta-analysis of 31 cohort studies involving 297 685 participants (22 639 incident T2DM cases) with dietary intake assessment and 84 171 participants (18 458 incident T2DM cases) with biomarker measurements^(28)^. Higher dietary LA intake was associated with a 6 % lower risk of T2DM (RR 0·94; 95 % CI: 0·90, 0·99). In a dose-response analysis, each 5 % increment in energy from LA was associated with a 10 % lower risk of T2DM. The summary RR for incident T2DM per standard deviation increase in LA concentrations in blood compartments or adipose tissue was 0·85 (95 % CI 0·80, 0·90).

### Clinical evidence

The results of RCT are generally supportive of the protective effects of LA intake on metabolic changes relevant to developing and/or managing T2DM. For example, the results of a meta-analysis of 102 RCT that included 239 dietary intervention arms involving 4220 adults found that replacing 5 % of TDE from SFA with PUFA significantly lowered blood glucose, hemoglobin A1c, C-peptide and homeostatic model assessment-insulin resistance^(124)^. Furthermore, PUFA significantly improved insulin secretion capacity whether replacing carbohydrate, SFA or MUFA. Imamura *et al.* ^(124)^ concluded that, in comparison to carbohydrates, SFA, or MUFA, consistent favorable effects were seen with PUFA.

Three years later, Wanders *et al.* ^(125)^ published the results of a meta-analysis that included thirteen RCT and nineteen comparisons of plant-derived PUFA with controls that examined glucose metabolism and insulin resistance. In contrast to the analysis by Imamura *et al.* ^(124)^, PUFA did not significantly affect fasting glucose; however, PUFA lowered fasting insulin and homeostatic model assessment-insulin resistance. Finally, in contrast to the meta-analyses conducted by Imamura *et al.* ^(124)^ and Wanders *et al.*,^(125)^ a meta-analysis of ten parallel and twenty crossover RCT involving 1586 participants failed to find that replacing SFA with MUFA or PUFA had significant effects on insulin sensitivity. The authors noted that many of the trials were relatively short-term and that longer term studies evaluating glucose homeostasis are needed^(126)^.

In conclusion, epidemiologic and clinical trial evidence suggests that replacing SFA with PUFA may reduce the risk of developing T2DM and favorably affects metabolic changes related to diabetes.

## Biological relevance of the *n*-6:*n*-3 fatty acid ratio

The *n*-6:*n*-3 fatty acid ratio is calculated by summing all *n*-6 PUFA in circulation or the diet divided by the sum of all *n*-3 PUFA. The *n*-6:*n*-3 ratio was first popularised by Simopoulos^(127)^ and Lands^(128)^ in the 1990s. The concept is based on the established competition between the two essential fatty acids – LA and ALA – for metabolism by desaturase enzymes in the synthesis of long-chain *n*-3 PUFA, especially arachidonic acid (AA; 20:4*n*-6), eicosapentaenoic acid (EPA; 20:5*n*-3) and docosahexaenoic acid (DHA; 22:6*n*-3). Since, ALA conversion to EPA and DHA can be accelerated by reducing intake of LA, the ‘balance’ idea appeared reasonable. As appreciation of the health importance of *n*-3 PUFA grew throughout the 1990s, ways to increase tissue levels of EPA and DHA took on new importance, which led to the vilification of *n*-6 PUFA and a high dietary *n*-6:*n*-3 PUFA ratio.

However, there are several flawed assumptions about the use of the dietary *n*-6:*n*-3 ratio as a metric, which severely limit its utility. For example, the components of the *n*-6:*n*-3 ratio are rarely defined. Generally, three *n*-6 PUFA are in the diet (LA, AA and trace amounts of gamma-linolenic acid) and another four *n*-6 metabolites are present in the blood (dihomo-gamma linolenic acid, adrenic acid, eicosadienoic acid and *n*-6 docosapentaenoic acid). In comparison, there are four dietary *n*-3 PUFA, mostly ALA but also EPA, *n*-3 docosapentaenoic acid and DHA. In the blood, DHA is the most prevalent. Depending on the analytical methods used, sometimes all 11 PUFA are used in the calculation of the *n*-6:*n*-3 ratio, whereas, in some cases, the calculation will include far fewer. Since it is rarely reported which PUFA are included, the ratio is non-specific.

In addition, identical ratios can be calculated from different absolute amounts of individual PUFA. A diet containing 15 g of *n*-6 PUFA and 1 g of *n*-3 PUFA has the same ratio (15:1) as a diet containing 5 g of *n*-6 PUFA and 0·33 g of *n*-3 PUFA. A specific dietary *n*-6:*n*-3 ratio can be achieved by an almost infinite set of dietary patterns, some of which could be deficient in both fatty acid classes. Thus, dietary recommendations should not be made based on the *n*-6:*n*-3 ratio, but on the absolute intake of *n*-3 and *n*-6 PUFA.

Furthermore, the notion that *n*-6 PUFA are pro-inflammatory and that *n*-3 PUFA are anti-inflammatory^(129,130)^ contrasts with the new understanding that such broad categorisation of *n*-6 and *n*-3 PUFA is far too simplistic^(131)^ and has little to no direct support from studies in humans^(37,132,133)^. In fact, evidence shows that higher LA levels are associated with reduced inflammatory status^(134–136)^. Higher inflammatory status is observed when EPA and DHA levels are low^(137)^ (i.e. the ratio is high), but the problem is not the presence of *n*-6 PUFA, rather the relative absence of *n*-3 PUFA. Even if evidence indicated that *n*-6 PUFA and AA are pro-inflammatory, this would not suggest the same of LA since the assumption that lowering LA intake will lower tissue levels of AA is not supported by available evidence^(138)^. Tracer studies have shown that <0·2 % of dietary LA is converted to AA^(139)^.

Finally, oxylipins, lipid mediators produced from metabolism of both *n*-6 and *n*-3 PUFA, may explain the diverse and complex effects of this class of fatty acids^(140)^. In some cases, *n*-6 oxylipins have similar beneficial roles as *n*-3 oxylipins, although they are not as potent^(140)^. The bulk of the evidence now supports the cardiovascular benefits of both *n*-6 and *n*-3 PUFA. There are many conceptual limitations to the dietary *n*-6:*n*-3 ratio that render it clinically and biologically irrelevant. As observed by Lucas, ‘What is the usefulness of the ratio of *n*-6 to *n*-3, which is good divided by good?’^(141)^.

## Guidance for health professionals on oils high in UFA

Dietary guidance for general health and CVD prevention focuses on dietary patterns rather than single foods or nutrients^(9–12,53–55)^. Recommendations are based on evidence from RCT and observational studies conducted in various populations that has demonstrated healthful dietary patterns are associated with lower risk of CVD^(142–144)^. Several healthful dietary patterns have been described, such as the Mediterranean, Dietary Approaches to Stop Hypertension, Healthy U.S.-style and plant-based patterns. Common elements to healthful dietary patterns include an emphasis on minimally processed foods, including fruits, vegetables, whole grains, healthful sources of proteins (e.g. fish, seafood, beans, lentils, tofu and other soy foods, nuts and seeds) and non-tropical, liquid plant oils in place of solid (more saturated) fats. Healthful dietary patterns are also low in processed meats, refined grains, added sugars, salt, SFA and *trans* fatty acid^(9–12,53–55,145)^.

Vegetable oils containing PUFA can be routinely used in cooking with some considerations for proper use. Concerns surrounding the use of PUFA-containing oils include UFA oxidation and the production of other potentially harmful by-products^(21)^. The smoke (burning) point is important for determining the type of oil to use in cooking (i.e. high *v*. low heat). Several research groups and/or organisations have provided smoke point measurements for a variety of edible oils^(146–149)^. Several oils with moderate to high amounts of PUFA have a high smoke point, such as avocado, peanut, canola and sunflower oils, and can be used with higher heat cooking without adverse consequences (Table 4).

**Table 4.** Smoke point of vegetable oils*

Oil type	Celsius	Fahrenheit	Reference
Avocado	197	387	142
Canola	236–256	457–492	142, 143, 145
Coconut	175–196	347–385	142, 143, 145
Corn	230–235	446–455	143, 145
Cottonseed	215–232	419–450	143, 145
Extra-virgin olive	207	405	142
Grapeseed	268	514	142
High-oleic canola	240	464	145
High-oleic sunflower	244	471	145
Low linolenic soybean	237	458	145
Mid oleic sunflower	211	412	145
Olive (refined)	190–208	374–406	142, 143
Palm	254	489	143, 145
Palm hard fraction (IV-35)	230	446	145
Palm olein (IV-57)	230	446	145
Peanut	225–230	437–446	143, 145
Rice bran (high oryzanol)	222	432	142, 145
Rice bran	229–237	444–459	145
Sesame	227	441	144
Soyabean	225–240	437–464	143, 145
Sunflower	255	491	142

* Values represent typical smoke points for commercially available edible oils based on tests conducted on various oil batches at various laboratory facilities. The values do not represent a statistically valid mean or indicate the range of values from a single source for each of the oils.

In contrast, oils like extra-virgin olive oil have a lower smoke point and should be used for low-heat cooking only or in recipes that do not require cooking (e.g. salad dressing) (Table 4). Deep frying with low smoke point oils should be avoided because the temperature of oils during deep frying exceeds the recommended temperature (180 °C or 356° F) to avoid the production of harmful by-products. The repeated use of frying oil (i.e. 8–10 frying cycles), especially at higher cooking temperatures and with intermittent heating and cooling of the oil, results in the increased production of free fatty acids, SFA and *trans* fatty acid^(150–155)^. Although the repeated use of frying oils is a potential concern with commercial establishments, reusing frying oil may be a practice in some populations (e.g. Asian Indians)^(152)^. Thus, consumers should receive education to avoid reusing oils when cooking.

Proper storage of vegetable oils is important to prevent them from going rancid and developing an unpleasant smell or flavour. Heat, light and exposure to oxygen increase the risk of oils turning rancid^(156)^. Proper storage of vegetable oils includes keeping them in a dark place (e.g. pantry or cupboard), reducing air exposure by placing the cap on tightly between uses and keeping oils at room temperature. Oils should not be stored on a countertop near a stove or oven because this will increase both light and heat exposure. For freshness and quality, it is recommended that vegetable oils be used within 6–12 months of purchase, if stored properly, and within 3–5 months after opening, if stored properly^(157)^.

## Summary and conclusions

For many decades, the relationship between dietary fat intake and health has been rigorously investigated with an emphasis on CVD. In many countries, current dietary guidelines do not include recommendations to limit total fat intake, rather, the focus is on the type of fat to consume. Strong and consistent evidence demonstrates that higher intake of PUFA is associated with lower risk of incident CVD. In addition, replacement of SFA with PUFA reduces the risk of CAD events and CVD mortality. Less data are available for the relationship between MUFA intake and CVD, although the existing evidence suggests higher intake of MUFA from plant sources is associated with lower risk of CVD. In alignment with observational evidence, results from clinical trials show higher intake of PUFA and MUFA from plant-sources improves major CVD risk factors, including levels of atherogenic lipids and lipoproteins. Observational and clinical evidence also suggests that diets higher in UFA reduce the risk of developing T2DM and increase insulin sensitivity.

One topic of debate within the fatty acid field is the clinical relevance of the dietary ratio of *n*-6 to *n*-3 fatty acids. At one point, the consensus was that a high ratio was considered to be harmful because LA and ALA compete for desaturation enzymes, and because metabolites of *n*-6 AA were considered to be pro-inflammatory. However, the utility of this ratio has been rejected by health agencies throughout the world. One reason for this rejection is the recognition that the *in vivo* conversion of LA to AA is negligible, and another reason is that some metabolites of AA exert anti-inflammatory effects. There is also concern that, because of its multiple double bonds, LA intake could promote oxidative stress. However, clinical trial evidence shows the intake of *n*-6 PUFA does not increase markers of inflammation or oxidative stress. Nevertheless, because carbon–carbon double bonds are susceptible to oxidation, high *n*-6 PUFA oils can become rancid if improperly stored. Therefore, consideration of how these oils are stored and how they are used in cooking, especially frying, is important.

In conclusion, authoritative health and scientific organisations recommend intake of *n*-6 PUFA-containing vegetable oils, including seed oils, as part of healthful dietary patterns. Specifically, vegetable oils rich in UFA should be consumed instead of rich sources of SFA, such as butter, tallow, lard, palm and coconut oils, duck fat and ghee.
