# Perspective on the health effects of unsaturated fatty acids and commonly consumed plant oils high in unsaturated fat – CORRIGENDUM

**DOI:** 10.1017/S0007114524002915

**Published:** 2024-10-28

**Authors:** Kristina S. Petersen, Kevin C. Maki, Philip C. Calder, Martha A. Belury, Mark Messina, Carol F. Kirkpatrick, William S. Harris

In the article by Petersen et al. entitled “Perspective on the health effects of unsaturated fatty acids and commonly consumed plant oils high in unsaturated fat” funding was mistakenly listed as coming from the USA Canola Association rather than the U.S. Canola Association.
